# HTLV-I Tax inhibits innate antiviral signaling via NF-κB-dependent induction of SOCS1

**DOI:** 10.1186/1742-4690-8-S1-A191

**Published:** 2011-06-06

**Authors:** Soratree Charoenthongtrakul, Qinjie Zhou, Noula Shembade, Nicole S Harhaj, Glen N Barber, Edward W Harhaj

**Affiliations:** 1 Department of Microbiology and Immunology, The University of Miami, Miller School of Medicine, Miami, FL, 33136, USA

## 

The human T cell leukemia virus type I (HTLV-I) inhibits host antiviral signaling pathways although the underlying mechanisms are unclear. Here we found that the HTLV-I Tax oncoprotein induced the expression of SOCS1, an inhibitor of interferon signaling. Tax required NF-κB, but not CREB, to induce the expression of SOCS1 in T cells. Furthermore, Tax interacted with SOCS1 in both transfected cells and in HTLV-I transformed cell lines. Although SOCS1 is normally a short-lived protein, in the presence of Tax, the stability of SOCS1 was greatly increased. Accordingly, Tax enhanced the replication of a heterologous virus, vesicular stomatitis virus (VSV), in a SOCS1-dependent manner. Surprisingly, Tax required SOCS1 to inhibit RIG-dependent antiviral signaling, but not the interferon-induced JAK/STAT pathway. Inhibition of SOCS1 by RNA-mediated interference in the HTLV-I transformed cell line MT-2 reduced HTLV-I replication and p19Gag levels. Taken together, our results reveal that Tax inhibits antiviral signaling, in part, by hijacking an interferon regulatory protein.

